# Cervical spine arthritis as initial manifestation in Juvenile Idiopathic Arthritis: a multicenter retrospective study

**DOI:** 10.1186/s12969-025-01161-9

**Published:** 2025-11-10

**Authors:** Estefania Quesada-Masachs, Jaime de Inocencio, Juan Carlos Nieto González, Indalecio Monteagudo Sáez, Sara Murias Loza, Marta Medrano San Ildefonso, Joan Calzada Hernández, Inmaculada Calvo Penadés, Rocío Galindo Zabala, Mireia Lopez Corbeto, Jordi Antón

**Affiliations:** 1 Division of Rheumatology, Department of Medicine, University of Miami Miller School of Medicine, Diabetes Research Institute. 1450 NW 10th Avenue, Room 5014, Miami, FL 33136 USA; 2 Pediatric Rheumatology Unit, Department of Rheumatology, Vall d’Hebron University Hospital, Barcelona Hospital Campus, Barcelona, Spain; 3 Pediatric Rheumatology Unit, Department of Pediatrics, University Hospital 12 de Octubre, Madrid, Spain; 4 Pediatric Rheumatology Unit, Department of Rheumatology, Gregorio Marañón University Hospital, Madrid, Spain; 5 Pediatric Rheumatology Unit, Department of Pediatrics, University Hospital Central of Asturias, Oviedo, Spain; 6 Department of Rheumatology, Miguel Servet University Hospital, Zaragoza, Spain; 7 Department of Pediatric Rheumatology, Hospital Sant Joan de Déu, Barcelona, Spain; 8 Department of Pediatric Rheumatology, La Fe University and Polytechnic Hospital, Valencia, Spain; 9 Sección Reumatología Pediátrica, UGC Pediatría, Hospital Regional Universitario de Málaga, Málaga, Spain; 10 Department of Public Health and Maternal and Child Health, Complutense University of Madrid, Madrid, Spain

**Keywords:** Juvenile Idiopathic Arthritis, Cervical, Spine, Treatment, TNFi, anti-TNFα

## Abstract

**Objective:**

To describe the clinical characteristics, diagnostic findings, treatment, and outcomes of patients with Juvenile Idiopathic Arthritis (JIA) whose initial manifestation was cervical spine arthritis.

**Methods:**

This retrospective multicenter study reviewed medical records of JIA patients who presented with cervical spine arthritis as the first and only symptom of the disease. Data collected included clinical presentation, imaging findings at diagnosis and follow-up, age at onset, JIA subtype, disease activity, treatment strategies, and clinical outcomes.

**Results:**

Nine patients were identified, five of whom were classified as having oligoarticular-persistent JIA. The mean age at symptom onset was 4.98 ± 3.08 years, with an average diagnostic delay of 6.98 ± 9.78 months. The most common presenting symptom was torticollis (88.9%), followed by limited cervical range of motion (77.8%) and neck pain (55.6%). Cervical MRI was performed in all cases, demonstrating C1–C2 synovitis in every patient and subluxation in four. Initial treatments included nonsteroidal anti-inflammatory drugs (NSAIDs), glucocorticoids, and methotrexate. Seven patients required escalation to biologic therapy with tumor necrosis factor inhibitors (TNFi), and one underwent surgical arthrodesis.

**Conclusions:**

Cervical spine arthritis may be the sole initial manifestation of JIA and should be considered in children with persistent torticollis. Early MRI evaluation is critical for timely diagnosis and monitoring. The frequent need for TNFi therapy highlights the potentially aggressive nature of this presentation and the importance of early recognition and intervention.

## Introduction

Juvenile Idiopathic Arthritis (JIA) is the most common chronic rheumatic disease in childhood and a leading cause of both short- and long-term disability [1]. Although JIA remains a clinical diagnosis, studies have shown that physical examination alone has limited reliability in children, even when performed by experienced clinicians [2]. This highlights the critical role of imaging techniques, particularly for assessing joints that are difficult to evaluate through physical examination.

Cervical spine involvement in JIA is not uncommon as a late complication of the disease, especially in the polyarticular and systemic JIA rather than the oligoarticular subtype [3–8]. Furthermore, radiological involvement of the cervical spine in JIA has been reported with a wide range of prevalence (5–80%) [4, 7, 8]. Comparisons across prevalence studies are hindered by heterogeneity in the methods used, variations in JIA subtypes (some studies only focused on polyarticular JIA), different length of observation periods, and different treatment approaches, particularly between the pre- and post-biologic era. Clinically, cervical spine involvement in JIA may range from mild symptoms—such as headaches or neck pain—to more severe manifestations, including torticollis, stiffness, restricted range of motion, or signs of cervical myelopathy. However, it is often asymptomatic [8]. Current treatment guidelines consider cervical spine involvement a poor prognostic factor and classify it as a high-risk joint in JIA [9, 10].

Cervical spine involvement is often not recognized in the early stages of JIA. In fact, it is a rare initial presentation and is exceptionally uncommon as the sole manifestation of the disease [11]. As a result, the frequency, clinical features, and outcomes of these patients remain poorly defined. The objective of this study was to describe the clinical characteristics and outcomes of nine JIA patients who presented with cervical arthritis—confirmed by imaging—as the first and only manifestation at disease onset.

## Materials and methods

A multicenter retrospective study was conducted. All pediatric rheumatologist members of the Spanish Society for Pediatric Rheumatology (*Sociedad Española de Reumatología Pediátrica*,* SERPE*) were invited to include their patients in the registry utilized for this study.

Patients who met the International League of Associations for Rheumatology (ILAR) criteria for Juvenile Idiopathic Arthritis (JIA) and presented with cervical spine involvement as the sole manifestation of the disease were included in the study [12]. Clinical data including demographic information, disease characteristics, imaging findings, treatments received, and clinical outcomes were extracted from medical records. Clinical outcomes were assessed at the last follow-up and included: number of swollen, painful, and limited joints, levels of acute phase reactants, and disease activity assessment [1] (inactivity, remission [13], minimal [14], moderate and high disease activity [15]). This study was performed in accordance with the Vall d’Hebron University Hospital Institutional Review Board (code number PR(AG)185/2015). Informed consent was obtained from all participants/legal guardians.

A descriptive statistical analysis was performed. Data is presented as mean ± standard deviation for continuous variables and numbers (percentages) for categorical variables.

## Results

### Clinical characteristics

Eight Spanish hospitals participated in the registry, and a total of nine Caucasian patients were included. The main clinical characteristics are summarized in Table 1. All patients tested negative for both rheumatoid factor and anti-cyclic citrullinated peptide (anti-CCP) antibodies. It is noteworthy that seven of the patients were ≤ 5-year-old at the time of onset and diagnosis. The mean delay from symptom onset to JIA diagnosis was 6.98 ± 9.78 months (range: 1–33 months). The most common initial symptoms of cervical arthritis were torticollis (8/9), limited range of motion (7/9), and neck pain (5/9). Over the course of the disease, six patients (66.7%) developed peripheral arthritis, and three developed chronic anterior uveitis—unilateral in one case and bilateral in two. Notably, three patients (33.3%) did not experience involvement of any other joints during follow-up.

**Table 1:** Demographic and main clinical characteristics of JIA patients

Patient	Sex	ILAR [12] Classification	ANA / HLA-B27	Age at initial cervical symptoms^#^, y	Age at JIA diagnosis, y	Time until other joint arthritis, mo	Other joints affected	Uveitis
1	M	Polyarticular RF Neg	Neg / Neg	2.3	2.5	4	Elbows, wrists	No
2	F	Oligoarticular persistent	Neg / Neg	4.2	4.4	-	-	No
3	F	Oligoarticular persistent	Neg / Neg	5.4	5.8	11	Knee	Yes
4	F	Oligoarticular persistent	Neg / Neg	8.9	11.6	-*	-*	No
5	M	Undifferentiated	Pos / Neg	4.0	4.4	6	Knee	No
6	F	Oligoarticular persistent	Pos / Neg	3.8	4.1	-	-	Yes
7	F	Polyarticular RF Neg	Neg / Neg	1.9	2.0	1	Ankles, PIP, MCP	Yes
8	F	Oligoarticular persistent	Pos / Neg	3.2	3.5	3	Knee	No
9	M	Undifferentiated	Pos / Pos	11.1	11.7	2	Hip	No

Abbreviations: ANA, antinuclear antigen; F, female; HLA, human leukocyte antigen; ILAR, International League of Associations for Rheumatology; M, male; mo, months; MCP: metacarpophalangeal joint; m, months; Neg, negative; Pos, positive; PIP: proximal interphalangeal joint; RF, rheumatoid factor; y, years

*That patient had no documented history of arthritis or pain in any other joint. However, at last follow-up the patient had bilateral limited range of motion of both temporomandibular joints. The underlying cause of this limitation has not been confirmed at the time of publication

^#^As “initial cervical symptoms” all patients presented torticollis, limited range of motion and/or neck pain. No other symptoms were reported

### Imaging features

**Fig. 1:**
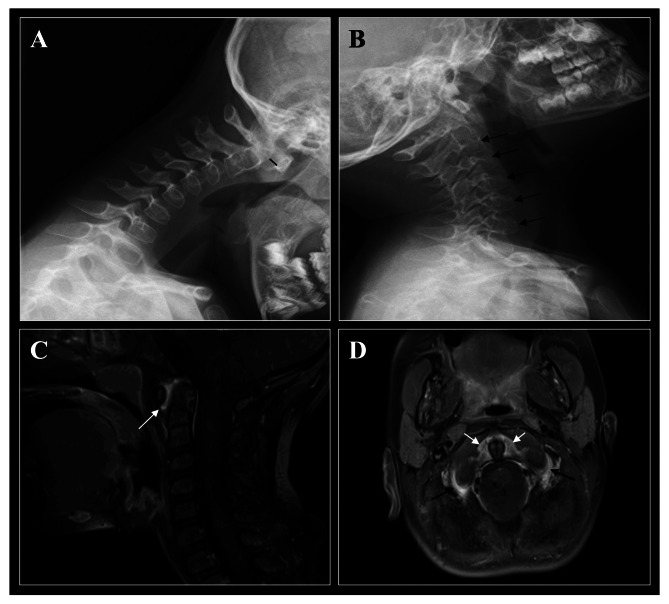
Representative images of the cervical spine involvement in a 4-year-old patient with oligoarticular JIA. **(A/B)** Lateral-view radiographs of the cervical spine at diagnosis (flexion/extension). **(A)** Cervical spine in flexion shows anterior atlantoaxial subluxation of 5.6 mm (black line). **(B)** Cervical spine in extension shows diffuse demineralization at C2-C6 vertebral bodies (black arrows). **(C/D)** MRI of the cervical spine of the same patient at diagnosis, both images of T1 sequences with fat saturation and with a contrast medium. **(C)** Sagittal section of the cervical spine shows an intense contrast enhancement and synovial hypertrophy in the atlanto-axial joint (white arrow). **(D)** Axial section of the cervical spine shows an intense contrast enhancement and synovial hypertrophy in the atlanto-axial joint in the pre- and paradental space (white arrows) and contrast enhancement in the lateral masses (both right and left) of the atlanto-axial joint (black arrows)

A plain radiograph (X-ray) was obtained as the initial imaging study in all patients (Fig. 1A, B). Abnormal findings were observed in four of the nine patients, including anterior atlanto-axial subluxation in three cases and nonspecific irregularities in the shape of the C2 and C3 vertebrae in one. Magnetic resonance imaging (MRI) was subsequently performed in all patients, revealing C1–C2 synovial pannus formation in all cases (Fig. 1C, D). Additional MRI findings included interapophyseal joint synovitis in 2 out of 9 patients and bone marrow edema in the C2, C3, and C4 vertebrae in one patient. MRI confirmed the presence of C1–C2 instability and anterior atlanto-axial subluxation in the three patients previously identified by radiography.

Computed tomography (CT) was performed in three patients, two of whom showed structural lesions, including erosions and atlanto-axial subluxation. One patient who underwent both MRI and CT presented vertebral erosions accompanied by bone edema. Bone scintigraphy was conducted in two patients—one at diagnosis and the other three months after disease onset. The first showed increased tracer uptake in the C1 and C2 vertebrae, while the second exhibited uptake only in the left knee, with no cervical spine involvement.

### Treatment

All patients were initially treated with non-steroidal anti-inflammatory drugs (NSAIDs), but responses were either absent or only partially effective. Seven patients (77.8%) received concomitant oral glucocorticoids (GC), with prednisone-equivalent doses ranging from 0.2 to 0.5 mg/kg/day. One patient achieved remission after two months of GC therapy, which was sustained even after discontinuation.

Methotrexate (MTX) was initiated in seven patients. Although all reported partial clinical improvement, none achieved remission. Consequently, treatment was intensified: one patient received combination therapy with MTX and leflunomide, while the remaining six were treated with the tumor necrosis factor inhibitor (TNFi) etanercept (ETN). Of these, three received ETN in combination with MTX, and three as monotherapy due to minor adverse events or lack of efficacy with MTX. Additionally, one patient received ETN monotherapy without prior MTX exposure.

Although ETN effectively controlled cervical arthritis, two patients developed uveitis during treatment and were switched to adalimumab, with favorable clinical responses. Overall, seven of the nine patients (77.8%) required biologic therapy with TNFi agents to achieve satisfactory disease control. One patient underwent cervical spine surgery (C1–C2 arthrodesis) four months after diagnosis due to a high risk of medullary compression. Supportive treatments included physical therapy in six patients and cervical spine collars in five.

### Clinical outcome

At the last follow-up visit (mean follow-up duration: 3.75 ± 1.63 years), acute phase reactants were within normal limits in all patients. Seven out of nine patients met the clinical criteria for remission of JIA. Among the two remaining patients, one fulfilled the criteria for minimal disease activity, primarily due to mild ocular inflammation and the other had a moderate active disease.

On physical examination, none of the patients had active, swollen, or painful joints. However, three patients showed persistent limited range of motion: two in the cervical spine, and one each in the elbow, temporomandibular joint, and hip.

## Discussion

Diagnosing Juvenile Idiopathic Arthritis (JIA) can be particularly challenging when cervical spine involvement is the sole initial manifestation, given the broad differential diagnosis of neck complaints in children and the variable clinical presentation [12]. Cervical arthritis is also often underdiagnosed during the disease course, as it may be minimally symptomatic or even asymptomatic. Previous studies have emphasized the importance of radiological assessment at disease onset and throughout follow-up—especially in polyarticular JIA—regardless of the presence of symptoms [16].

However, cervical spine involvement as the *first* and *only* manifestation of JIA is exceedingly rare. In one previous series, all 15 children with cervical arthritis had peripheral joint involvement at diagnosis [7], a key difference from our cohort in which none had other joints affected at diagnosis. To date, only 13 clinical cases have been published describing cervical spine arthritis as the initial and exclusive presentation of JIA [17–26]. To the best of our knowledge, our series represents the largest cohort of such cases published to date.

Notably, most patients in our cohort were classified as having oligoarticular-persistent JIA, in contrast with previous reports where cervical spine arthritis as an initial manifestation was more frequently described in systemic, polyarticular, or enthesitis-related arthritis subtypes [7, 8]. The predominance of oligoarticular JIA in our cohort may be reflecting the underlying epidemiological characteristics of the patient population in our country [27]. Although seven of our patients were aged ≤ 5 years at diagnosis, the mean age at JIA diagnosis in our patients (5.6 years) was consistent with previously reported national data [27]. In addition, three of our nine patients (33.3%) developed chronic anterior uveitis—unilateral in one and bilateral in two—despite initial presentation limited to the cervical spine. Chronic anterior uveitis was previously described in another oligoarticular case with cervical arthritis as a unique manifestation of JIA [23]. This underscores the need for early ophthalmologic screening in patients with cervical arthritis, even when the overall disease appears limited.

Torticollis was the most frequent presenting symptom in our cohort (88.9%), whereas limited range of motion and neck pain were also common. Torticollis is typically a benign, self-limiting condition in children—90% of cases have a trivial cause [25]—yet our findings suggest that persistent torticollis, particularly beyond six weeks, should prompt evaluation for JIA [28]. Interestingly, in another published cohort, torticollis was rare (1/15 patients), possibly due to earlier diagnosis because those patients had other joints affected and treatment or differences in disease severity [7]. It is plausible that torticollis may correlate with more advanced or active cervical involvement.

Radiographic abnormalities were present in 46% of our patients, including anterior atlantoaxial subluxation, which was later confirmed by MRI. MRI identified C1–C2 synovitis in all patients, as well as additional findings such as interapophyseal synovitis and bone marrow edema in selected cases. This confirms the superior sensitivity of contrast-enhanced MRI for early detection of cervical spine arthritis and its complications [6, 16, 29]. While radiographs can help identify structural instability, MRI remains the imaging modality of choice for evaluating synovitis, effusion, bone edema, and spinal cord relationships. However, no validated scoring system currently exists for assessing cervical spine arthritis by MRI in children [2, 30, 31].

Therapeutically, our data show that monotherapy with methotrexate was insufficient to achieve remission in any of the treated patients, despite partial clinical improvement. Consequently, 7 out of 9 patients (77.8%) required escalation to biologic therapy with tumor necrosis factor inhibitors (TNFi). One patient achieved remission with glucocorticoids alone. These findings align with previous case reports where early TNFi therapy was associated with marked reductions in cervical inflammation and improved clinical outcomes [7]. Thus, cervical spine involvement—even in the context of an otherwise limited disease—may warrant early initiation of TNFi therapy.

Our study has limitations, most notably its retrospective design and small sample size. However, due to the rarity of cervical spine arthritis as the initial and exclusive presentation of JIA, we report the largest such cohort to date. Although all members of the Spanish Society of Pediatric Rheumatology (SERPE) were invited to participate, reporting was voluntary; thus, some cases may have gone unreported. Systemic JIA cases were not included, as all with cervical involvement also had systemic features at onset. The absence of a validated standard for grading cervical arthritis severity in pediatric patients also limits direct comparison across studies and between clinical centers.

## Conclusion

In summary, we describe a cohort of JIA patients whose disease initially and exclusively affected the cervical spine. Torticollis was the most frequent presenting symptom, highlighting the importance of considering JIA in the differential diagnosis of prolonged torticollis or restricted neck motion. Most patients were classified as oligoarticular and a significant proportion developed uveitis and required TNFi therapy, suggesting a potentially more severe disease course than expected. While radiographs can be useful as an initial imaging test for identifying instability, MRI remains the preferred tool for early detection and monitoring of cervical synovitis. Early recognition of cervical arthritis in JIA is crucial, as it can influence prognosis, guide treatment decisions, and help prevent long-term structural damage.

## Data Availability

No datasets were generated or analysed during the current study.

## Declarations

### Author information (optional)

Estefania Quesada-Masachs is a pediatric rheumatologist and currently serves as a Research Assistant Professor at the University of Miami Miller School of Medicine. Her areas of expertise include type 1 diabetes, in-situ protein expression in human tissue, musculoskeletal imaging, juvenile idiopathic arthritis and early-onset autoimmune diseases.
